# Effect of glycerol, n, n-dimethylformamide and n-methyl-2-pyrrolidone on rabbit sperm stored at 4 °C and 16 °C

**DOI:** 10.21451/1984-3143-AR2018-0100

**Published:** 2019-11-14

**Authors:** Paula Domingo, Maite Olaciregui, Noelia González, Ignacio De Blas, Lydia Gil

**Affiliations:** 1 Universidad de Zaragoza, Facultad de Veterinaria, Área de Obstetricia y Reproducción Animal, Departamento de Patología Animal, España; 2 Universidad de Zaragoza, Facultad de Veterinaria, Área de Salud Animal, Departamento de Patología Animal, España

**Keywords:** cryoprotectant, dimethylformamide, n-methyl-2-pyrrolidone, glycerol, rabbit sperm preservation

## Abstract

Artificial insemination with cooled semen is the most common practice in rabbit farms and any improvement on it helps to increase the efficiency and productivity of rabbit meat farms. Therefore, the aim of this study was to assess whether different cryoprotectant agents (CPA) as glycerol, N, N-Dimethylformamide (DMF) and N-Methyl-2-Pyrrolidone (NMP) can improve cooled rabbit sperm quality stored at 4ºC and 16ºC. Sperm samples were diluted with INRA 96^®^ (Extender A), INRA 96^®^ with 6% glycerol (Extender B) or 6% DMF (Extender C) or 6% NMP (Extender D) respectively and stored at 4ºC and 16ºC. Samples were then analysed at 4, 24, 48 and 72 hours after refrigeration by integrated sperm analysis system (ISAS^®^), eosin-nigrosin stain (vitality), hypo-osmotic swelling test (HOS test) and acrosome integrity test. Extender C showed higher percentage of motility, vitality and HOS test than extender B and D (*p<0.05*). Whereas sperm quality decreased over time (*p<0.05*), data showed that the addition of DMF kept the motility and sperm plasma membrane integrity after 24 hours of storage better than other diluents. These results suggest that the addition of DMF to INRA 96^®^ exerts a protective effect on the membrane of spermatozoa improving seminal quality.

## Introduction

Spermatozoon survival is affected by storage temperature and extender (Carluccio et al., 2004). Currently, the most common temperature used to preserve rabbit sperm in farms is around 16 °C due to the fertility is considerably lower after 48 hours of storage (López and Alvariño, 1998; Roca et al., 2000; Rosato et al., 2006; Johinke et al., 2014). Likewise, the extender affects sperm quality. Many extenders (López and Alvariño, 2000; Carluccio et al., 2004) and substances (Trejo et al., 2013; Johinke et al., 2014; Sarıözkan et al., 2014) have been evaluated in order to preserve semen the most time possible for carry it to another farms. Nevertheless, Carluccio et al. (2004) demonstrated that INRA 96® kept rabbit sperm quality better than others extenders even though INRA 96® was made specifically for the preservation of stallion sperm.

Moreover, because of rabbit sperm characteristics (high activation energy and low water permeability coefficient), cryoprotectant agents (CPA) with lower molecular weight and higher permeability, such as amides or methyl groups, are suitable to be used (Curry et al., 1995; Mocé and Vicente, 2009). N, N-Dimethylformamide (DMF) is an amide solvent used in chemical reactions. Previous studies in boar (Malo et al., 2012), canine (Futino et al., 2010; Lopes et al., 2009; Mota et al., 2011), goat (Bezerra et al., 2011) and fowl sperm (Chalah et al., 1999) demonstrated that DMF is not better CPA than glycerol. However, stallion sperm (Alvarenga et al., 2005; Olaciregui et al., 2014; Pukazhenthi et al., 2014) diluted with DMF as an alternative to glycerol showed better results. N-Methyl-2-Pyrrolidone (NMP) is also an amide solvent commonly used in chemical reactions as an alternative to DMF. But unlike DMF, NMP has not been studied before for storage sperm samples.

On the other hand, glycerol is the main CPA used to preserve domestic or wild animal sperm (Curry et al., 1995). Nevertheless, glycerol has not been the first CPA of choice to preserve rabbit semen due to its toxicity, which may result in osmotic stress, protein denaturation, alteration of actin interactions and induction of protein-free membrane blister that leads to get worse fertility (Alvariño, 1993; Gilmore et al., 1995; Okuda et al., 2007; Iaffaldano et al., 2012).

The lack of previous studies using DMF or NMP as a CPA in rabbit sperm preservation and the supposed toxicity of glycerol, leads to study more these CPA. The aim of this study was to assess the quality of cooled rabbit sperm stored at 4 °C and 16 °C and diluted with INRA 96® supplemented with glycerol, DMF or NMP. Moreover, evaluate the effect of storage time during 72 hours and decide whether glycerol, DMF or NMP can be used as CPA on cooled rabbit semen preservation.

## Methods

### Chemicals

Unless noted otherwise, all chemicals were from Panreac Quimica S.L.U (Barcelona, Spain).

### Animals, semen collection and processing

The study was performed following approval by the Veterinary Ethical Committee of University of Zaragoza. The care and use of animals were performed according to the Spanish Policy for Animal Protection RD1201/05, which meets the European Union Directive 86/609 on the protection of animals used for experimental and other scientific purposes.

Rabbit sperm samples were collected from eight sexually mature bucks previously selected from a commercial AI centre (Técnicas Cunícolas S.A., Zaragoza, Spain) and used as semen donors. Males were housed in individual cages with 12 hours of light and 12 hours of darkness at a room temperature of 22-24 °C and a relative humidity of 60-70%. All rabbits were fed a commercial pellet diet according to their reproductive condition and fresh water was provided *ad libitum*.

Rabbit sperm samples were collected using artificial vagina (IMV Technologies, L’Aigle, France). After semen collection, any gel plug was removed and a macroscopic analysis was performed assessing the colour and the volume of the sample. The first microscopic analysis of the motility in the farm was made as well. Only ejaculates with white colour, more than 0,2ml and good wave motion (at least 85% of motility) were used for the research.

All ejaculates were pooled with the purpose of eliminate individual differences, thereafter were divided in four fractions and each one was diluted with a different extender: INRA 96® (IMV Technologies, L’Aigle, France) (Extender A) as control, and INRA 96® supplemented with 6% glycerol (Extender B), 6% DMF (Extender C) or 6% NMP (Extender D).

Subsequently, each semen sample was placed into two Eppendorf tube in order to store each sample at 4 °C and at 16 °C. Samples were cooled progressively from 37 °C to 16 °C and 4 °C in a time period among 90 and 120 minutes (Mocé and Vicente, 2009) and finally they were stored.

### Evaluation of spermatozoa

Sperm motility, vitality, membrane integrity and acrosome integrity were assessed at 4, 24, 48 and 72 hours after collection for both temperature storage and all extenders.

#### Sperm motility and kinematics

Sperm motility and kinematics parameters were evaluated by ISAS® software (PROISER R+D, Valencia, Spain) following the default setting specifically for rabbits. Five microliters were placed on a slide and covered with 20 × 20mm coverslip. Five fñields were randomly captured at ×10 magnification by phase-contrast microscope. Up to 200 frames per second were acquired selecting particles with an area of between 10 and 70µm^2^. The cut-off values used to determine sperm velocity were 10<slow<25<medium<50<rapid (µm/s). The linearly motile sperm were deviated <45% from a straight line. The analyses provided information about the percentage of motile spermatozoa (MOT, %), curvilinear velocity (VCL, µm/s), straight-line velocity (VSL, µm/s), average path velocity (VAP, µm/s), linearity (LIN=VSL/VCL, %), straightness (STR=VSL/VAP, %), wobble (WOB=VAP/VCL, %), amplitude of lateral head displacement (ALH, µm) and beat cross frequency (BCF, Hz).

#### Vitality

Eosin-nigrosin stain was used to evaluate the vitality of the spermatozoa following the protocol described by Björndahl et al. (2003). According to the stain penetration trough the damaged membrane the dead spermatozoa had red or dark pink heads (stained) and the live spermatozoa had white heads (unstained) (WHO, 2010).

#### Sperm plasma membrane integrity

Hypo-osmotic swelling test (HOS test) was performed following the protocol established by Jeyendran et al. (1984). Ten microliters of semen were mixed with 90µl of HOS test solution (100 mM of Sodium Citrate) and kept at 37 °C at least 30 minutes. Subsequently 100 µl of glutaraldehyde 2% was added to the sample for fix it. Each sample was analysed by phase-contrast microscope at x400 magnification. Spermatozoa with intact membranes allowed an influx of water inside the cell giving place to swollen spermatozoa with coiling tail (Amorim et al., 2009; WHO, 2010).

#### Acrosome integrity

Acrosome integrity test is based on the fixation of the spermatozoa. Ten microliters of semen was immediately fixed in 90µl of glutaraldehyde 2% solution, right after each sample was analysed by phase-contrast microscope at x1000 magnification (Pursel and Johnson, 1974). Acrosomes were differentially categorized into two classes: intact acrosome (normal apical ridge) and damaged acrosome (damaged apical ridge and/or missing apical ridge).

### Statistical analysis

The study was replicated three times. Data were analysed using IBM SPSS Statistics 23 for Windows. Results were expressed as mean ± SEM. The effect of the extender, storage temperature and time of storage on the studied parameters were analysed by ANOVA. Duncan's post-hoc test was used to evaluate the effect of the extender and time of storage. The interactions among the extender used, storage temperature and time elapsed were assessed using GLM procedure. The level of significance was set at *p<0.05*.

## Results

**Figure 1:**
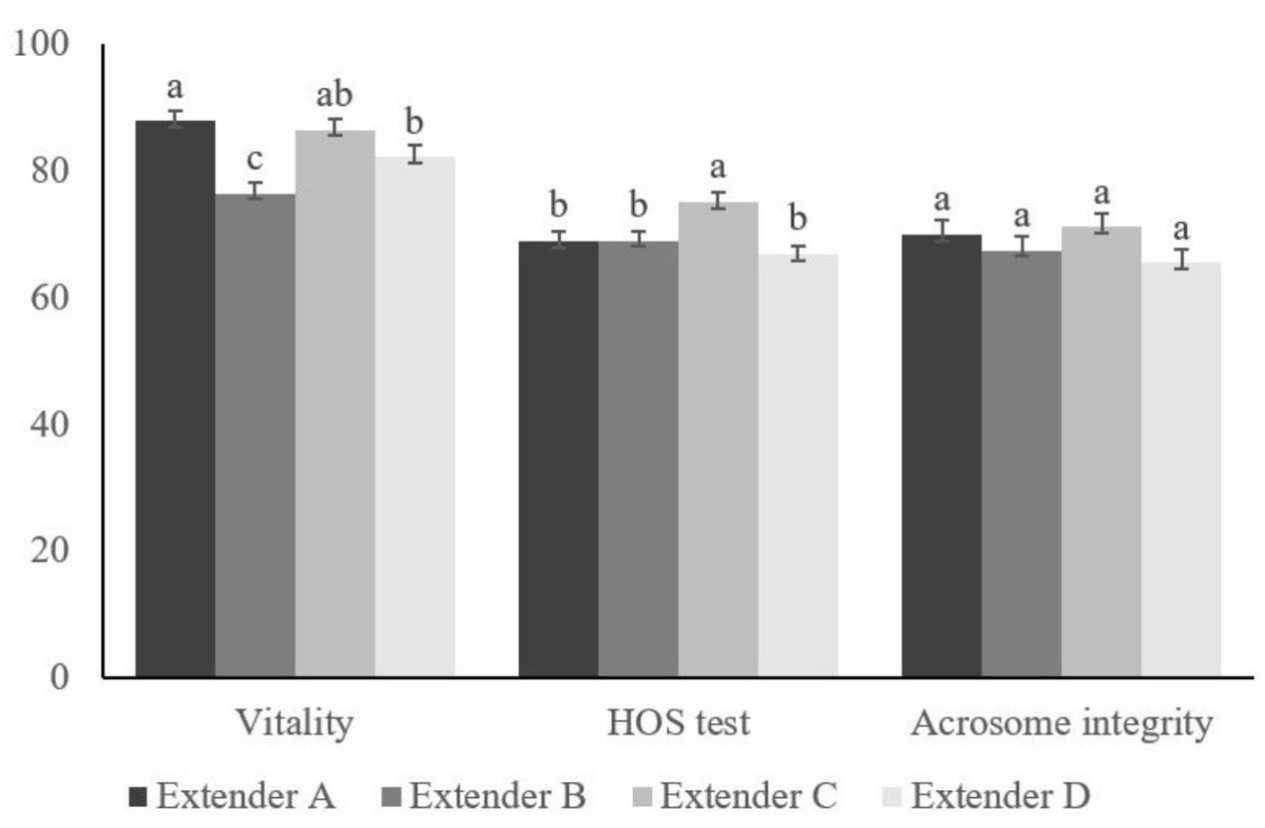
Percentage of vitality, HOS test and acrosome integrity of rabbit spermatozoa diluted with extender A, extender B, extender C and extender D from the 16 °C and 4 °C groups. Data are mean ± SEM. Different letters within each diagnostic test denote statistical differences (p<0.05).

The effect of the extender played an important role in seminal preservation, in fact, all parameters studied were significantly significant (*p<0.05*), excluding acrosome integrity parameter (Table 1 and Figure 1). When the samples were diluted with extender C, the data obtained were similar to control samples (extender A), moreover extender C got higher percentage (*p<0.05*) of MOT, vitality and HOS test. A few worse results of MOT, vitality and HOS test were obtained by extender D, though the worst results were observed in samples diluted with extender B. Other kinematic parameters were studied, showing extender A greater results on velocity and trajectory.

**Table 1:** Effect of the extender on kinematic parameters determined by ISAS. Different letters within each column indicate statistically significant differences (*p<0.05*). Data are mean ± SEM.

	MOT	VCL	VSL	VAP	LIN	STR	WOB	ALH	BCF
	**(%)**	**(µm/s)**	**(µm/s)**	**(µm/s)**	**(%)**	**(%)**	**(%)**	**(µm)**	**(Hz)**
**Extender A**	59.9 ± 2.4^a^	73.7 ± 2.4^ab^	26.7 ± 1.1^a^	43.9 ± 1.4^a^	35.1 ± 1.1^a^	59.1 ± 1.0^bc^	58.1 ± 1.0^a^	2.9 ± 0.1^b^	7.3 ± 0.3^b^
**Extender B**	35.4 ± 2.4^c^	66.9 ± 2.3^b^	22.1 ± 1.1^b^	36.1 ± 1.4^b^	34.9 ± 1.9^a^	61.1 ± 1.0^b^	55.9 ± 1.0^a^	3.1 ± 0.1^b^	7.6 ± 0.3^ab^
**Extender C**	59.3 ± 2.2^a^	75.5 ± 2.3^a^	21.5 ± 1.1^bc^	37.2 ± 1.3^b^	29.5 ± 1.0^b^	58.0 ± 1.0^c^	50.3 ± 1.0^b^	3.4 ± 0.1^a^	8.4 ± 0.3^a^
**Extender D**	47.5 ± 2.2^b^	56.7 ± 2.3^c^	18.9 ± 1.1^c^	28.3 ± 1.3^c^	33.9 ± 1.0^a^	65.9 ± 1.0^a^	51.5 ± 1.0^b^	3.0 ± 0.1^b^	8.3 ± 0.3^a^
***p***	*0.001*	*0.001*	*0.001*	*0.001*	*0.001*	*0.001*	*0.001*	*0.001*	*0.001*

**Figure 2:**
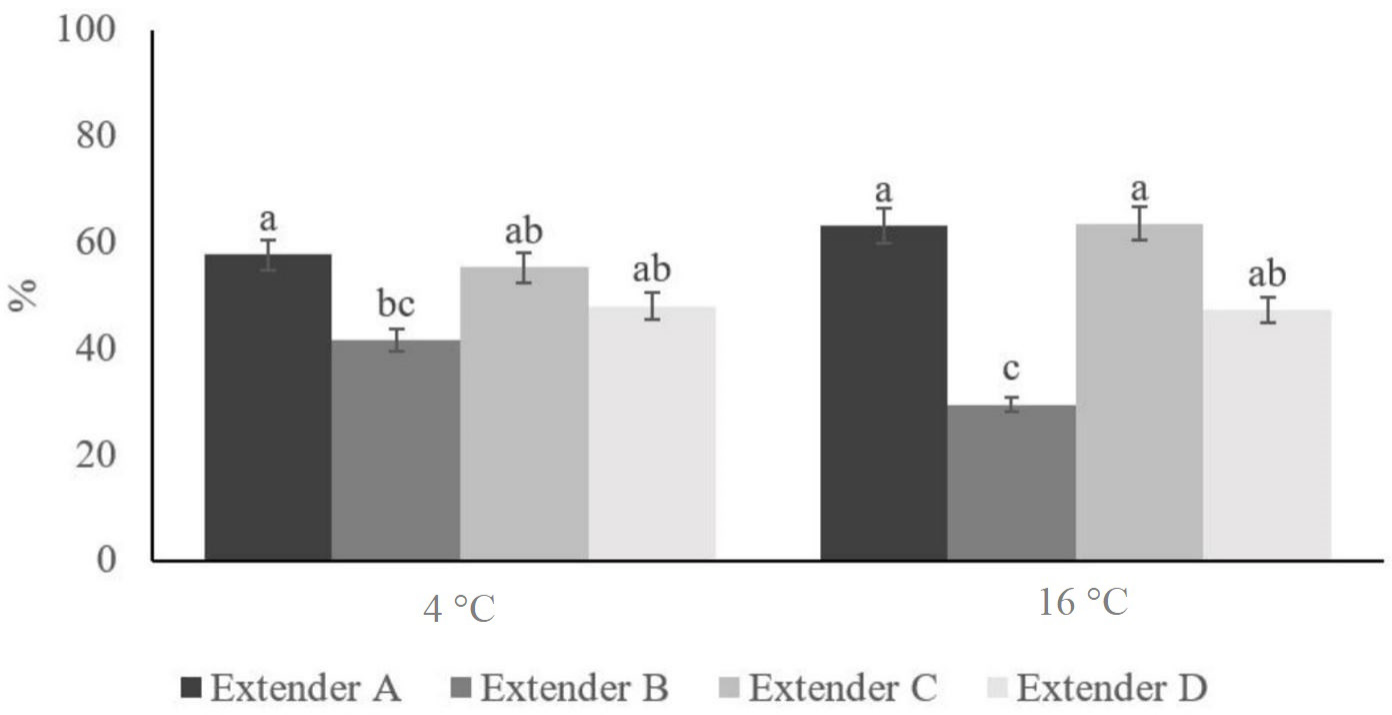
Effect of the temperature on the motility parameter of rabbit spermatozoa diluted with extender A, extender B, extender C and extender D. Data are mean ± SEM. Different letters within each diagnostic test denote statistical differences (p<0.05).

When the effect of storage temperature on cooled rabbit sperm was analysed no significant differences were observed on MOT, kinematic parameters, vitality, HOS test and acrosome integrity. With the exception of BCF parameter (*p<0.001*), which was higher when sperm samples were stored at 4 °C (8.307 ± 0.189 Hz) instead of 16 °C (7.475 ± 0.203 Hz). In spite of no statistically differences were observed between storage samples at 4 °C or 16 °C, it should be noted that studying thoroughly the interaction between the diluents and the temperature statistically differences (*p=0.027*) on MOT were shown (Figure 2).

Regarding all sperm parameters studied, sperm quality decreased over storage time (*p<0.05*) (Table 2). The percentage of total sperm motility dropped off from 64.7 ± 2.3% at 4 hours after collection to 44.5 ± 2.3% passing 48 hours. Nevertheless, the sharpest decline of motility was from 72 hours of storage (28.6 ± 2.6% of motile spermatozoa). The vitality of spermatozoa was kept till 72 hours after storage, from 87.5 ± 1.8% to 75.3 ± 1.7% of live spermatozoa. Almost all kinematic parameters and acrosome integrity shown a considerable decline after 24 hours of storage. Lastly, sperm plasma membrane was started to damage as of 48 hours.

**Table 2:** Effect of time of storage on MOT, kinematic parameters, vitality, HOS test and acrosome integrity. Different letters within each column indicate statistically significant differences (*p<0.05*). Data are mean ± SEM.

Time of storage	MOT	VCL	VSL	VAP	LIN	STR	WOB	ALH	BCF	Vitality	HOS test	Acrosome integrin
(hours)	(%)	(µm/s)	(µm/s)	(µm/s)	(%)	(%)	(%)	(µm)	(Hz)	(%)	(%)	(%)
4	64.7 ± 2.3^a^	76.9 ± 2.3^a^	31.6 ± 1.0^a^	46.4 ± 1.3^a^	41.3 ± 1.0^a^	68.0 ± 1.0^a^	60.2 ± 1.0^a^	3.1 ± 0.1^b^	9.3 ± 0.3^a^	87.5 ± 1.8^a^	79.2 ± 1.4^a^	79.7 ± 2.2^a^
24	60.8 ± 2.3^a^	79.7 ± 2.3^a^	22.5 ± 1.0^b^	39.7 ± 1.3^b^	28.7 ± 1.0^c^	57.1 ± 1.0^b^	50.0 ± 1.0^c^	3.5 ± 0.1^a^	8.4 ± 0.3^b^	84.8 ± 1.9^a^	75.3 ± 1.4^a^	70.8 ± 2.2^b^
48	44.5 ± 2.3^b^	64.5 ± 2.3^b^	18.3 ± 1.0^c^	31.2 ± 1.3^c^	29.4 ± 1.0^c^	59.2 ± 1.0^b^	49.2 ± 1.0^c^	3.1 ± 0.1^b^	7.9 ± 0.3^b^	85.5 ± 1.7^a^	68.8 ± 1.4^b^	66.8 ± 2.2^b^
72	28.1 ± 2.6^c^	48.5 ± 2.6^c^	15.5 ± 1.2^c^	26.0 ± 1.5^d^	33.9 ± 1.1^b^	60.1 ± 1.1^b^	56.1 ± 1.1^b^	2.8 ± 0.1^b^	5.7 ± 0.3^c^	75.3 ± 1.7^b^	56.7 ± 1.5^c^	57.0 ± 2.2^c^
*p*	*0.001*	*0.001*	*0.001*	*0.001*	*0.001*	*0.001*	*0.001*	*0.023*	*0.001*	*0.017*	*0.001*	*0.001*

**Figure 3:**
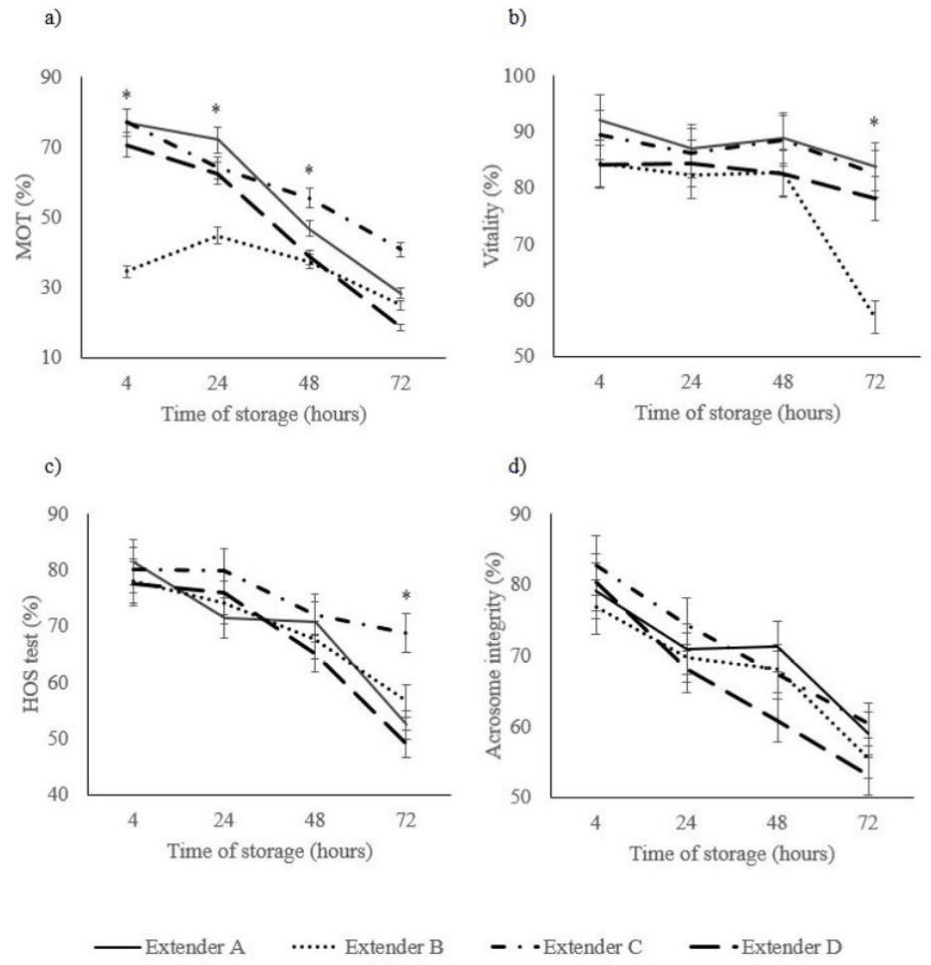
Percentage of MOT, vitality, HOS test and acrosome integrity of rabbit spermatozoa at 4, 24, 48, and 72 hours from the four extender (A-D) groups. Data are mean ± SEM. Means marked with an asterisk within each time point denote statistical differences (p<0.05).

The interaction between the extender and the hours that elapsed since the collection of the samples are presented in Figure 3. All the parameters studied, except acrosome integrity, were statistically significant (*p<0.05*) showing that as time goes by, semen quality decreases.

Extender A obtained the highest MOT up to 24 hours, but after that period of time the addition of DMF to INRA 96® offered higher MOT. Extender C obtained the best data on MOT at 72 hours (40.8 ± 4.6%). MOT parameter was similar for extender C and D until 24 hours of storage, thereafter MOT dropped reaching less than 20% at 72 hours after collection. Extender B obtained the worst MOT in relation to other extenders (*p<0.05*) (Figure 3a).

The percentage of alive spermatozoa obtained with extender A was similar than extender C during 72 hours (Figure 3b). Extender B or D showed similar values till 48 hours of storage, after 48 hours of storage the vitality of spermatozoa diluted with extender B came down from 82.6 ± 3.5% at 48 hours to 56.9 ± 3.5% at 72 hours of storage (*p<0.05*).

The evaluation of the damage of the sperm plasma membrane is synthesise in Figure 3c. As it is shown, extender C offered better protection on the sperm plasma membrane, observing statistically differences (*p<0.05*) after 72 hours of storage.

## Discussion

It is known that the addition of CPA to an extender is performed with the goal of protect the sperm from cryodamage, which causes devastating consequences in sperm survival during sperm cooling process. Though also, CPA, such as glycerol, could have a toxic effect on sperm (membrane destabilization, protein and enzyme denaturation, osmotic stress, alteration of actin interactions and induction of protein-free membrane blister) directly related to the concentration used and the time of cell exposure (Alvariño, 1993; Gilmore et al., 1995; Okuda et al., 2007; Iaffaldano et al., 2012). Interestingly, this study on rabbit sperm showed same results as previous studies in stallion sperm preservation (Olaciregui et al., 2014; Pukazhenthi et al., 2014): the addition of DMF maintained cooled rabbit sperm quality better than glycerol. In addition, samples diluted with INRA 96® plus DMF got, along with samples diluted with INRA 96®, the best sperm quality during cooled sperm preservation. Surprisingly, sperm quality decreased when samples were diluted with INRA 96® plus NMP. Reminding of rabbit sperm characteristics, the most appropriate solvents to use in rabbit sperm preservation should have low molecular weight and high permeability, such as amides (Curry et al., 1995; Mocé and Vicente, 2009). Therefore, the differences found between DMF and NMP could be owing to their molecular weights and densities. Despite DMF and NMP are both an amide solvents, NMP has higher molecular weight (99.13g/mol) and density (1.028g/cm^3^) than DMF (73.09g/mol of molecular weight; 0.944g/cm^3^ of density). These small differences in their composition might suggest that are responsible for NMP not being a good CPA for storage cooled rabbit sperm. Anyhow, further studies should be carried out to determine the optimal concentration of NMP.

On the other hand, as expected glycerol got the worst data due to its toxicity previously demonstrated by other authors (Alvariño, 1993; Gilmore et al., 1995; Okuda et al., 2007), not getting nor 30% of motile rabbit sperm storing it at 16 °C.

Previous studies have shown the ability of rabbit sperm to resist environmental stress. Because of the droplets and vesicles present in rabbit sperm; they are rich in cholesterol and phospholipids, assisting to modulate the membrane fluidity and in this way to withstand greater cold shock (Sparr et al., 2002; Castellini et al., 2006; Mocé and Vicente, 2009). Moreover, the high cholesterol:phospholipid ratio of rabbit sperm plasma membrane allows the ability to fertilize oocytes from cooling sperm at 4 °C is similar to fresh sperm (Mocé and Vicente, 2009). In the present study on cooled rabbit sperm, we demonstrated that there are no differences between storage rabbit sperm at 4 °C or 16 °C when INRA 96® is the extender used, contrarily other study published by Johinke et al. (2014) which using Tris-citric-acid extender with two different concentrations of glucose, obtained better results when stored rabbit sperm at 15 °C instead of 5 °C. Considering this, we can appreciate that the extender and CPA play an important role, moreover INRA 96® extender can be used satisfactorily among 4 °C and 16 °C.

As expected, in this study the sperm quality of all the samples decreased throughout storage time as other authors previously reported (López and Alvariño, 1998; Roca et al., 2000), showing after 48 hours a considerable drop on MOT parameter. Nevertheless previous studies demonstrated that MOT is not the only good parameter to evaluate sperm quality, other parameters related with MOT such as VCL, VSL, VAP, LIN, STR, WOB, ALH and BCF are also useful to predict male *in vitro* fertility (Liu et al., 1991; Hagen et al., 2010). To date, all kinematic parameters in rabbit spermatozoa have never been studied previously by other authors. Surprisingly, in this study we can appreciate that almost all kinematic parameters only were maintained till 24 hours, unlike vitality parameter which hardly showed a decline during 72 hours of storage. Moreover, even though the quality of all the samples decreased throughout storage time, data showed that the addition of DMF kept the motility and sperm plasma membrane integrity after 24 hours of storage better than other extenders.

Summarising, these results demonstrated that the addition of DMF to INRA 96® diluent exerts a protective effect on the membrane of spermatozoa and therefore it improve seminal quality. Moreover NMP could not be used to replace DMF considering that worse results were obtained. Also we are in agreement with previous results reported for the toxicity of glycerol, which made it not a good cryoprotector (Alvariño, 1993; Gilmore et al., 1995; Okuda et al., 2007). Finally, the temperature of storage among 4 °C and 16 °C did not affect the quality of rabbit sperm diluted with INRA 96®.

## Abbreviations

CPA: Cryoprotectant agents

DMF: N, N-Dimethylformamide

NMP: N-Methyl-2-Pyrrolidone

AI: Artificial insemination

MOT: Percentage of motile spermatozoa

VCL: Curvilinear velocity

VSL: Straight-line velocity

VAP: Average path velocity

LIN: Linearity

STR: Straightness

WOB: Wobble

ALH: Amplitude of lateral head displacement

BCF: Beat cross frequency

HOS test: Hypo-osmotic swelling test
